# Complete Genome Sequence of Leuconostoc kimchii Strain NKJ218, Isolated from Homemade Kimchi

**DOI:** 10.1128/MRA.00367-19

**Published:** 2019-07-03

**Authors:** Ji Young Jung, Jin-Woo Jeong, Seung-Young Lee, Hyun Mi Jin, Hee Won Choi, Byung-Gon Ryu, Sang-Soo Han, Hye Kyeong Kang, Eu Jin Chung, Kyung-Min Choi

**Affiliations:** a Microbial Research Department, Nakdonggang National Institute of Biological Resources (NNIBR), Sangju-si, Gyeongsangbuk-do, South Korea; b Animal & Plant Research Department, Nakdonggang National Institute of Biological Resources (NNIBR), Sangju-si, Gyeongsangbuk-do, South Korea; c Strategic Planning Department, Nakdonggang National Institute of Biological Resources (NNIBR), Sangju-si, Gyeongsangbuk-do, South Korea; d Bioresources Industrialization Support Department, Nakdonggang National Institute of Biological Resources (NNIBR), Sangju-si, Gyeongsangbuk-do, South Korea; University of Maryland School of Medicine

## Abstract

Leuconostoc kimchii strain NKJ218 was isolated from homemade kimchi in South Korea. The whole genome was sequenced using the PacBio RS II and Illumina NovoSeq 6000 platforms. Here, we report a genome sequence of strain NKJ218, which consists of a 1.9-Mbp chromosome and three plasmid contigs. A total of 2,005 coding sequences (CDS) were predicted, including 1,881 protein-coding sequences.

## ANNOUNCEMENT

Kimchi, a representative of traditional Korean food, is made from the fermentation process by lactic acid bacteria (LAB), which are major members of the kimchi bacterial community, including the genera *Leuconostoc*, *Lactobacillus*, and *Weissella* (1–7). To understand the kimchi fermentation process and standardize the quality, many researchers focused on the isolation of LAB and analyses of their diverse metabolic capacities and functionalities (3, 7–9). Based on accepted results from previous studies, isolated kimchi LAB were placed under the spotlight because of their beneficial effects, such as immunomodulatory effects, protection against enteric pathogen infection, bioconversion activity, and probiotic activities (10–13). For these reasons, kimchi LAB are being used in functional products for human health and are increasingly used as microorganisms in animal feed additives. Leuconostoc kimchii, one of the major kimchi LAB, was studied as a kimchi starter for increasing its health benefits, and some reports showed several beneficial influences, including lipid metabolism regulation (14, 15). We isolated Leuconostoc kimchii NKJ218 from homemade kimchi collected in Gyeongsangbuk-do province (South Korea) and sequenced and analyzed its whole genome to gain better insight into its metabolic capacity and functionality.

The genomic DNA of strain NKJ218 was prepared from an overnight culture grown at 30°C in MRS broth (Difco) using a TruSeq DNA PCR-free kit (Illumina). To generate a high-quality genome assembly, both long-read and short-read sequencing platforms were applied. The whole genome was sequenced at Cosmo Genetech (Seoul, South Korea) using a combination of a Pacific Biosciences (PacBio) RS II single-molecule real-time (SMRT) sequencing platform with a 20-kb SMRTbell template library and an Illumina NovaSeq 6000 platform (2 × 101 bp) with an insert size of 550 bp. A total of 57,991 postfilter polymerase reads (558,601,397 bp, mean read length of 4,140 bp) were generated from PacBio sequencing, and the 87,750 high-quality subreads (557,079,326 bp, mean subread length of 6,348 bp) were produced with quality filtering (minimum polymerase read quality, 0.75; minimum polymerase read length, 50 bp) and adapter trimming using Hierarchical Genome Assembly Process 3 (HGAP3) within PacBio SMRT analysis 2.3.0 (16). This was followed by a preassembly process for the error correction of long subreads by mapping of the shorter subreads on the longer subreads (called seed reads) with a length cutoff of 11,721 bp using HGAP3 (16). After preassembly, 10,104 error-corrected long subreads (103,476,822 bp, mean read length of 10,241 bp) were generated and *de novo* assembled for making the draft genome sequence using HGAP3 (16). To gain a more accurate genome sequence, approximately 5,031.2 Gbp (2,523.86-fold coverage) with 49,813,426 paired-end reads were additionally generated from the Illumina NovaSeq 6000 platform, and raw sequencing data were used for the consensus genome polishing and error correction by mapping on the initial PacBio draft assembly with HGAP3 (16). The gene prediction and annotation were performed with the NCBI Prokaryotic Genomes Annotation Pipeline (PGAP) 4.8 (17). The SEED subsystem was used via the RAST server 2.0 for functional categorization of the predicted proteins (18).

The final high-quality genome assembly, which had a mean coverage of 213.81-fold and GC content of 37.78%, consisted of a circular chromosome (1,917,647 bp, 37.90% GC content), two circular plasmids designated pLKN1 (29,596 bp, 35.49% GC content) and pLKN2 (20,691 bp, 36.41% GC content), and a potential unnamed plasmid fragment (25,506 bp, 32.54% GC content). The genome assembly and annotation statistics are shown in Table 1. The NCBI PGAP predicted 1,881 protein-coding genes, 12 rRNA genes, 67 tRNA genes, 3 noncoding RNAs, and 42 pseudogenes. Rapid Annotations using Subsystems Technology (RAST) annotation with the RASTtk scheme and default parameters (18, 19) showed that carbohydrate metabolism-involved genes were enriched. Carbohydrate metabolism yielded 12.7% of all matches, and the read fractions of central carbohydrate metabolism (32.5%), monosaccharide (18.8%), disaccharide and oligosaccharide (12.8%), and fermentation (17.1%) within the carbohydrate category were high. The strain NKJ218 genome contains 42 carbohydrate-active enzyme (CAZyme) genes, as predicted by HMMER searches (E value, <1e-15; coverage, >0.35) in dbCAN (20), including 19 genes encoding glycoside hydrolases (GHs), 3 genes encoding carbohydrate esterases (CEs), 19 genes encoding glycosyltransferases (GTs), and a polysaccharide lyase (PL), which are responsible for catalyzing the degradation or modification and potential utilization of carbohydrates. Also, the NCBI PGAP revealed the presence of several genes involved in bacteriocin production. Our systematic endeavor of genome sequencing, assembling, and functional annotation will provide an understanding of the whole metabolic and functional potential of Leuconostoc kimchii strain NKJ218.

**TABLE 1:** Summary of assembly and annotation statistics of Leuconostoc kimchii NKJ218

Genetic element	Genome size (bp)	GC content (%)	No. of coding sequences	No. of rRNAs	No. of tRNAs	Coverage (×)	GenBank accession no.
Chromosome	1,917,647	37.90	1,786	12	67	218.49	CP037939
Plasmid pLKN1	29,596	35.49	33	0	0	149.45	CP037938
Plasmid pLKN2	20,691	36.41	22	0	0	140.04	CP037937
Unnamed plasmid	25,506	32.54	40	0	0	68.40	CP037936
Total	1,993,440	NA^*a*^	1,881	12	67	NA	NA

a NA, not applicable.

### Data availability.

This genome sequence of Leuconostoc kimchii strain NKJ218 has been deposited in GenBank under the accession numbers CP037936, CP037937, CP037938, and CP037939, BioProject number PRJNA521423, and BioSample number SAMN10887832 (Table 1). The raw sequencing data have been deposited in the NCBI Sequence Read Archive (SRA) under the accession numbers SRR9076598 and SRR9076599.
